# Combined Effects of Visual Scanning Training and Electrical Stimulation During Walking on Activities of Daily Living in a Subacute Stroke Patient With Unilateral Spatial Neglect: A Case Study

**DOI:** 10.7759/cureus.85674

**Published:** 2025-06-09

**Authors:** Kodai Minami, Seigo Inoue, Yuto Goto, Kyohei Jumi, Kunitsugu Kondo, Katsuhiro Mizuno, Tatsunori Watanabe

**Affiliations:** 1 Department of Rehabilitation Medicine, Tokyo Bay Rehabilitation Hospital, Chiba, JPN; 2 Graduate School of Health Sciences, Aomori University of Health and Welfare, Aomori, JPN; 3 Department of Rehabilitation Medicine, Tokai University School of Medicine, Kanagawa, JPN; 4 Waseda Institute for Sport Sciences, Waseda University, Saitama, JPN

**Keywords:** activities of daily living, attention, hemiparesis, spatial neglect, transcutaneous electrical nerve stimulation

## Abstract

Interventions to improve activities of daily living (ADL) remain undeveloped for unilateral spatial neglect (USN). This case study examined the effects of combining visual scanning training (VST) and electrical stimulation during walking on ADL for USN. The patient was a 62-year-old right-handed man with dissection and occlusion of the right middle cerebral artery, who was transferred to our hospital on the 11th day from stroke onset. After approximately two months of conventional therapy, he was able to walk without an aid, but frequently overlooked objects and people on the left, requiring assistance. Thereafter, he underwent 14 days of combined VST with transcutaneous electrical nerve stimulation on his left posterior neck muscle during walking in addition to conventional physical therapy. The effectiveness of the intervention was assessed using the Behavior Inattention Test conventional subtest (BIT-c), Catherine Bergego Scale (CBS), and a computer-based assessment of top-down and bottom-up attention. Following the intervention, the BIT-c and CBS scores improved from 141 to 145 and from 11 to 2, respectively, and he rarely overlooked objects while walking. In addition, there was an improvement in attention automatically directed by external stimuli (bottom-up attention). These improvements were maintained at two-week follow-up. These findings suggest that the combined training during walking may have contributed to the improvement of ambulatory ADL in this stroke patient with USN, highlighting its potential relevance for functional independence in similar cases.

## Introduction

Unilateral spatial neglect (USN) is defined as a failure to detect or respond to stimuli in the space contralateral to a cerebral hemisphere lesion, which cannot be attributed to sensory or motor impairments [1]. It is commonly observed following right hemisphere damage and occurs in approximately 50% of patients with a right hemispheric stroke [2]. Clinically, USN reduces gait independence [3] and increases the risk of falls among hospitalized patients [4]. Furthermore, it can limit not only independence in activities of daily living (ADL), such as dressing and eating [5], but also social participation, such as outdoor walking [6] and resuming driving a car [7]. Therefore, developing effective interventions for USN is crucial for achieving successful ADL outcomes in rehabilitation medicine.

USN has been considered a disorder of the visuospatial attention network, which consists of the dorsal and ventral attention networks [8,9]. The dorsal attention network comprises neural connections between the superior parietal lobule, the intraparietal sulcus, and the frontal eye field, and is involved in top-down attention, enabling the voluntary allocation of attention to specific spatial locations and object characteristics. In contrast, the ventral attention network consists of neural connections between the temporoparietal junction and the middle and inferior frontal gyri and is associated with bottom-up attention, which allows for the detection of sudden stimuli and environmental changes. For example, our attention is unconsciously drawn to sudden sounds, bright lights, or moving objects. This function plays an important role in quickly detecting potential dangers or responding to significant changes. Thus, damage to the dorsal attention network leads to difficulty directing attention to the left side, whereas damage to the ventral attention network impairs the ability to detect unexpected changes on the left side. While dysfunction in each of these networks has been implicated in certain aspects of USN [8,10], their interaction is known to play a crucial role in the attentional network [8]. Specifically, excessive activation of top-down attention suppresses bottom-up attention, potentially delaying responses to sudden external stimuli. Conversely, when bottom-up attention is functionally dominant, top-down attention is weakened, hindering goal-directed behavior. In other words, these two networks dynamically switch depending on the situation's demands, maintaining a balanced interaction to prevent overreliance on either system. Therefore, considering the interplay between these two networks is essential when developing interventions for stroke patients with USN.

Top-down and bottom-up approaches have been proposed as treatments for USN [11]. The top-down approach enhances awareness of left space through visual, auditory, and linguistic cues, promoting voluntary spatial exploration. One example is visual scanning training (VST), in which patients search visual targets on both the right and left sides [12], thereby reinforcing attention toward the neglected space and improving spatial awareness. In contrast, the bottom-up approach integrates peripheral stimuli and adjusts the spatial coordinate system. This includes unilateral stimulation [13], prism adaptation [14], limb activation [15], and virtual reality [16]. Among these interventions, transcutaneous electrical nerve stimulation (TENS) applied to the left posterior neck [13,17,18] or the dorsum of the left hand [18] has been reported to improve performance in letter cancellation and copying tasks in patients with left USN. Furthermore, combining TENS with VST has been demonstrated to be more effective than either intervention alone [19-21]. For example, Polanowska et al. [19] investigated the combined effect of VST and TENS on USN and reported that, compared to VST alone, the combination significantly improved performance in line and star cancelation tasks. However, its impact on daily functional abilities, such as self-care and mobility, remains unclear, which limits its clinical applicability. Current evidence further suggests that the effectiveness of USN interventions on ADL remains unclear or limited [22,23].

Although conventional top-down and bottom-up approaches for USN are generally effective in alleviating neglect symptoms in most stroke patients, some individuals continue to exhibit persistent symptoms. These patients have been reported to be at a higher risk of falls [4], show reduced independence in ADLs [3,5], and require significantly longer hospital stays [5]. Meanwhile, task-oriented interventions targeting the upper extremities have been demonstrated to improve not only motor performance but also ADL functioning [24]. Therefore, investigating the combined effect of VST and TENS contributes to the development of a novel intervention strategy aimed at promoting independence in daily living among stroke patients with persistent neglect symptoms.

Accordingly, the purpose of this case study was to clarify the combined effects of VST and TENS on ADL in a stroke patient with USN. The effectiveness of the intervention was evaluated using a pre-post design. Since conventional pencil-and-paper tasks (e.g., line cancellation test) have been reported to lack sufficient sensitivity in detecting mild or subtle impairments [25,26], a recently developed licensed software operated via a touch-panel display (see details in Patient information) was used to assess both top-down and bottom-up attention [27,28].

## Case presentation

Patient information

**Figure 1:**
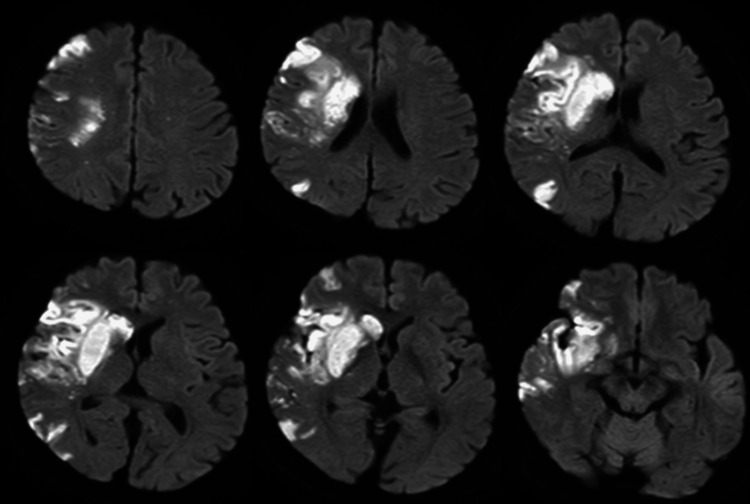
Brain images of the patient. Diffusion-weighted magnetic resonance images of the patient (day three from stroke onset).

A 62-year-old right-handed man with an acute onset of left hemiplegia and reduced consciousness was transported to an emergency department. Brain magnetic resonance imaging revealed dissection and occlusion of the right middle cerebral artery (Figure 1). He was transferred to our hospital on the 11th day from stroke onset.

At initial evaluation, the stroke impairment assessment set (SIAS) lower extremity (LE) motor function score was 8/15 points (moderate to severe paresis). Functionally, the patient had difficulty moving his legs voluntarily due to motor paralysis. The patient also exhibited moderate sensory dysfunction in his leg. The Berg Balance Scale (BBS) score was 15/56 points. The patient ambulated with a T-cane but required moderate assistance. The 10-meter walking speed (10MWS) and Timed Up and Go (TUG) test were 46.89 and 22.14 seconds, respectively. Cognitive assessments revealed that the Mini-Mental State Examination-Japanese (MMSE-J) score was 28/30 points while the Trail Making Test (TMT) parts A and B times were 137 and 241 seconds, respectively. USN was evaluated using the Behavior Inattention Test conventional subtest (BIT-c), a widely used pencil-and-paper test comprising six subscales: line bisection, line crossing, letter cancellation, star cancellation, figure and shape copying, and representational drawing. The patient was seated on a chair with a backrest, with both feet placed flat on the ground during testing. The BIT-c total score ranges from 0 to 146, with a score of 131 or lower indicating the presence of USN. The patient’s BIT-c score was 113/146 points, indicating USN. Additionally, there were instances in which the patient ignored verbal cues or stimuli from the left side, as well as occasions when the left upper limb had slipped off the chair's armrest. Furthermore, during walking, the patient not only dragged the left foot but also frequently failed to notice people or objects on the left side, requiring constant assistance.

The rehabilitation program during hospitalization included physical, occupational, and speech therapy, each provided for one hour per day (approximately seven times per week). Physical therapy focused on ankle range of motion exercises and standing and gait training. Occupational therapy included upper limb function exercises using a pegboard and an assist robot for left upper limb paralysis. Speech therapy primarily involved pencil-and-paper tasks such as line and letter cancellation tasks. After two months, the SIAS LE motor function improved to 12/15 points, with mild sensory improvement. The patient showed improvements in his leg coordination and was able to move his legs more smoothly. The BBS also improved to 51/56 points. The patient was able to walk without a T-cane, and his 10MWS and TUG improved to 8.60 and 11.46 seconds, respectively. The TMT parts A and B improved to 86 and 118 seconds, respectively. The BIT-c score increased to 141/146 points, suggesting improved USN on the pencil-and-paper test. Additionally, the patient was able to respond to verbal cues and stimuli from the left side, and the left upper limb no longer slipped off the chair's armrest. However, the patient continued to overlook objects and people on his left side during walking and always required assistance.

To assess USN in ADL, the Catherine Bergego Scale (CBS) was administered. This scale consists of 10 subscales: grooming, dressing, eating, mouth cleaning, gaze orientation, left limb awareness, auditory attention, collisions, spatial orientation, and finding personal belongings. Each item is scored on a four-point scale: 0 indicates no spatial neglect; 1 indicates mild neglect, with the patient consistently exploring the right hemispace first and moving hesitantly toward the left; 2 indicates moderate neglect, characterized by frequent omissions and collisions on the left side; and 3 indicates severe neglect, with the patient unable to explore the left hemispace. The CBS total score ranges from 0 to 30, with higher scores indicating greater severity of USN in ADL. The CBS score was 11/30 points, indicating moderate neglect symptoms [29]. The primary CBS deductions were collisions (3/3) and difficulty finding personal belongings (2/3), with neglect symptoms particularly evident in walking-related ADLs. Functionally, he demonstrated impaired awareness of his left side during ambulation, frequently overlooking pedestrians or obstacles and requiring verbal cues to avoid collisions.

In addition, @Attention (Creact Corp., Tokyo, Japan) [27,28], a recently developed licensed software operated via a touch-panel display, was used to assess impairments in top-down and bottom-up attention associated with USN. The software displays 35 black circular objects arranged in seven horizontal rows and five vertical columns on a white background. The patient sits in front of the monitor and selects the circular objects by touching them. There are two tasks: active and passive search tasks. The active search task assesses top-down attention and requires the patient to select and touch the circular objects in an arbitrary order using his index finger. Touched objects are displayed in green, repeatedly touched objects in red, and untouched objects remain black. On the other hand, the passive search task measures bottom-up attention and requires the patient to touch the circular object that flashes in a random order using his index finger. A timeout is set at five seconds, after which the object turns red if not touched. Objects touched before the timeout appear blue. The response time to each object is recorded and visualized as a spatial distribution. Two outcome measures were analyzed: the average reaction time (RT) for all objects (RT_mean_) and the left-right ratio (L/R ratio), which is calculated as the average RT for objects on the left divided by that for objects on the right, excluding the circular objects in the central vertical column. While RT_mean_ reflects general attention deficits, the L/R ratio indicates the presence of neglect symptoms. In the present case, no obvious abnormalities were observed in top-down attention, except for one instance of mistakenly selecting the object at the lower left twice (active search task). However, delayed RTs and omissions in responding to flashed stimuli on the left side indicated impaired bottom-up attention (passive search task).

Although the BIT-c scores improved following the conventional rehabilitation program, moderate neglect symptoms persisted in daily activities. Thus, VST combined with TENS during walking was added to the program. The timing of the aforementioned assessments conducted before this intervention (i.e., after the conventional rehabilitation program) was defined as pre-intervention. In this case study, the purpose, methods, and ethical considerations were explained to the patient, and written consent was obtained.

Intervention

**Figure 2:**
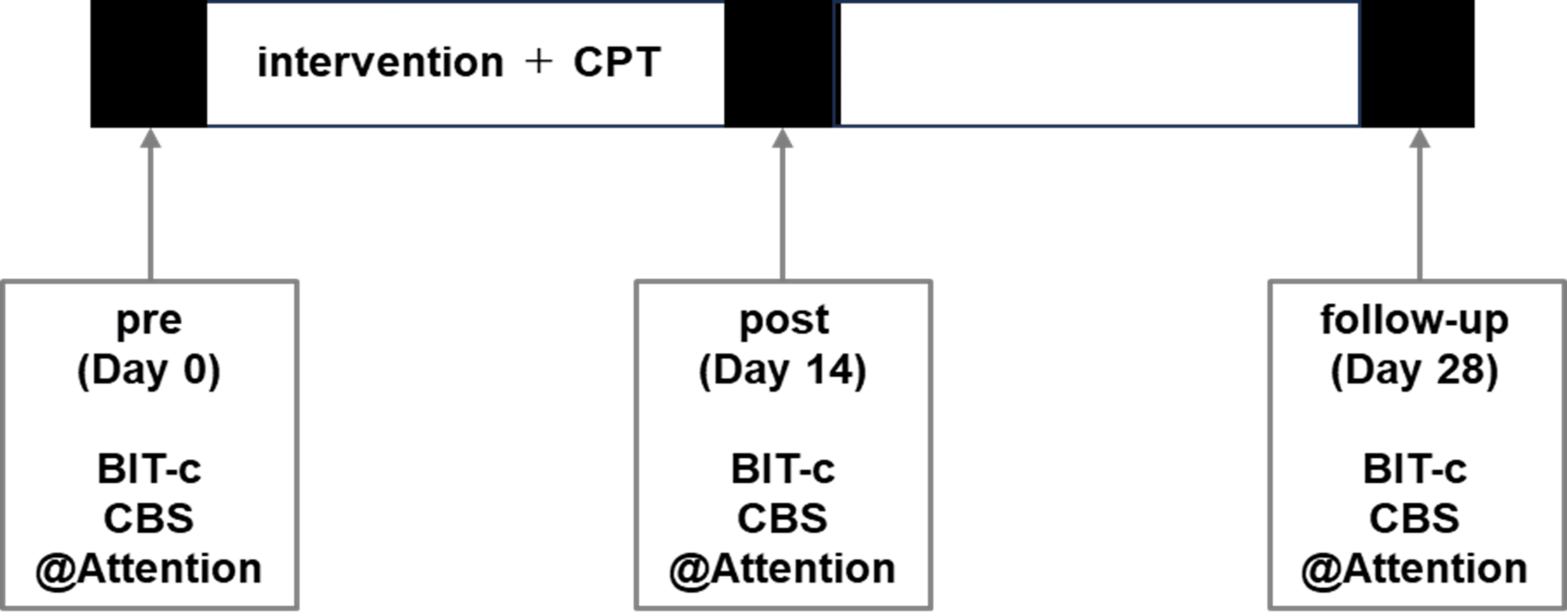
Intervention procedure. The effectiveness of the intervention was examined before and after the intervention and at a two-week follow-up using the Behavior Inattention Test conventional subtest (BIT-c), Catherine Bergego Scale (CBS), and @Attention. During the intervention, visual scanning training combined with electrical stimulation during walking was performed in addition to conventional physical therapy (CPT).

Intervention effects were verified using a pre-post design with a follow-up period (Figure 2). Evaluations were conducted before and after the intervention phase, and follow-up evaluations were performed two weeks after the final intervention to verify the carryover effect. In the intervention phase, the combination of VST with TENS during walking was performed in addition to conventional physical therapy.

**Figure 3:**
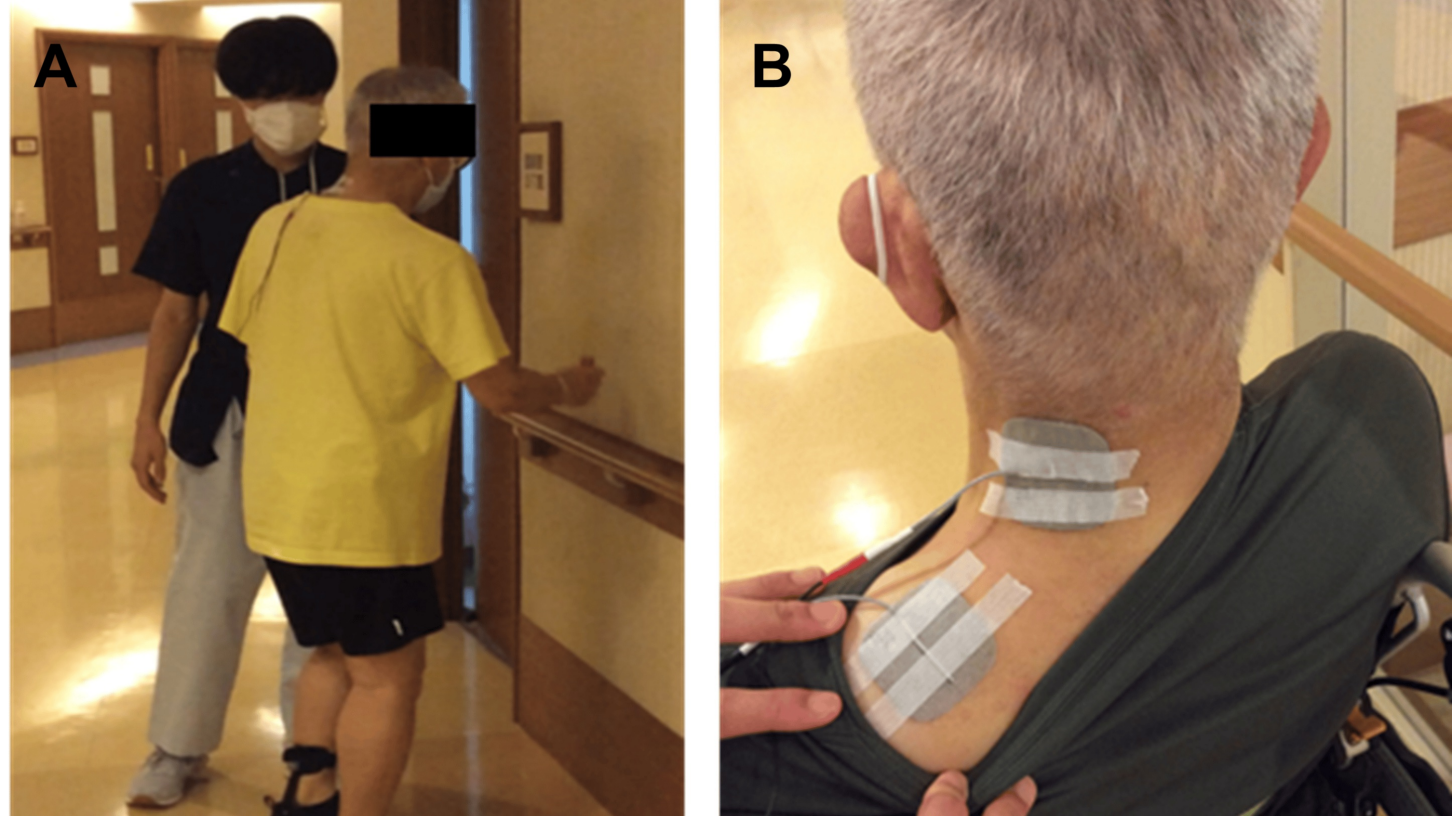
Combination of visual scanning training with electrical stimulation during walking. (A) Visual scanning training combined with electrical stimulation during walking. (B) Electrode placement on the left neck muscle.

The intervention involved the combination of VST with TENS during walking (Figure 3A). VST was conducted as a training in which the patient was asked to detect a total of 10 pegs and rings placed randomly on the left and right sides during walking. The number of correctly detected items was recorded, and the detection of all 10 items was considered the best performance. The task difficulty remained constant throughout the intervention. TENS was applied to the upper fibers of the left trapezius muscle simultaneously with the VST (Figure 3B). The ESPURGE (Ito Physiotherapy and Rehabilitation Co., Saitama, Japan) was used for TENS. Based on previous studies [19,20], the parameters were set with an output ranging from 11 to 16 mA, which is above the sensory threshold but below the motor threshold, a frequency of 100 Hz, and a pulse width of 100 μs, since we wanted to excite his sensory nerve without any pain. The combination of VST and TENS was conducted for approximately 20 minutes per day and continued for 14 days. To date, no adverse events have been reported in previous studies investigating the combination of VST and TENS. Blinding of the patient and assessors was not implemented due to the nature of the intervention, which made it infeasible. However, to minimize potential bias, standardized assessment protocols were employed, and efforts were made to ensure consistency across all evaluations.

Outcome measures

Outcomes were measured using the BIT-c, CBS, and @Attention before and after the intervention phase and at the two-week follow-up. The number of missing pegs and rings during VST was also measured during the intervention phase.

The total therapy duration was 2360 minutes, including 940 minutes of physical therapy (Table 1). There were no adverse events in this patient throughout the intervention.

**Table 1:** Therapy time during intervention phase (14 days).

Intervention phase	
Therapy time	
Physical therapy (min)	940
Occupational therapy (min)	720
Speech therapy (min)	700
Therapy frequency	
Physical therapy (days)	14
Occupational therapy (days)	14
Speech therapy (days)	13

**Figure 4:**
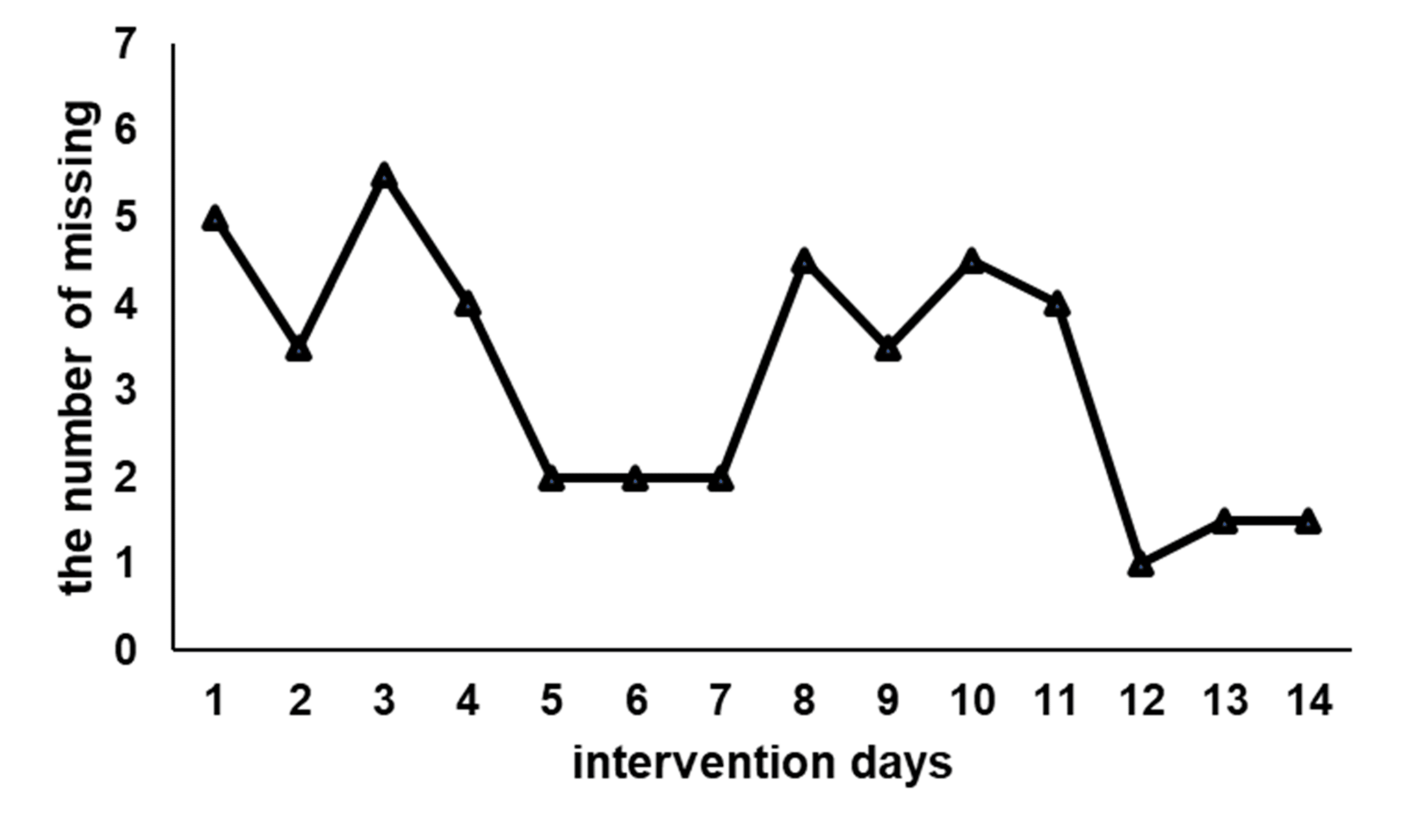
Results of visual scanning training. The Y-axis represents the number of missed pegs and rings, while the X-axis represents the intervention days. Although there were instances when the number of omissions increased during the intervention period, the number of missed pegs and rings tended to decrease as the intervention progressed. Functionally, the patient was able to walk safely without collisions after the intervention.

Figure 4 shows the number of missing pegs and rings during VST. The number of omissions tended to decrease as the intervention progressed.

Table 2 summarizes the clinical assessment results at the pre-intervention, post-intervention, and follow-up phases. The BIT-c scores were 141, 145, and 145 points at the pre-intervention, post-intervention, and follow-up phases, respectively. Notably, the number of omissions on the left side during BIT-c decreased after the intervention, indicating an improvement in neglect symptoms. Table 3 shows the CBS scores from the observational assessment, which were 11, 2, and 2, respectively, and the personal assessment, which were 2, 1, and 1, respectively. These results indicate that neglect symptoms in ADL improved to a mild level. He no longer overlooked people or objects on his left side and regained independent walking.

**Table 2:** Results of clinical tests in each phase. BIT-c, Behavior Inattention Test conventional; MMSE-J, Mini-Mental State Examination-Japanese; TMT, Trail Making Test.

	Pre-intervention	Post-intervention	Follow-up
BIT-c total score (0–146, cut off > 131)	141	145	145
Line bisection (0–9, cut off > 7)	9	9	9
Line cancellation (0–36, cut off > 34)	36	36	36
Star cancellation (0–54, cut off > 51)	52	53	53
Letter cancellation (0–40, cut off > 34)	37	40	40
Figure and shape copying (0–4, cut off > 3)	4	4	4
Representational drawing (0–3, cut off > 2)	3	3	3
MMSE-J (0-30)	28	-	29
TMT part A	86	-	83
TMT part B	118	-	116

**Table 3:** Results of the Catherine Bergego Scale (CBS). These results indicate that neglect symptoms in activities of daily living improved to a mild level. The patient no longer overlooked people or objects on his left side and regained independent walking.

	Pre-intervention	Post-intervention	Follow-up
Observational assessment			
CBS total (0-30)	11	2	2
Grooming	0	0	0
Dressing	1	0	0
Eating	0	0	0
Mouth cleaning	0	0	0
Gaze orientation	0	0	0
Left limb knowledge	2	1	0
Auditory attention	1	0	0
Collisions	3	0	1
Spatial orientation	2	0	0
Finding personal belongings	2	1	1
Personal assessment			
CBS total	2	1	1

**Figure 5:**
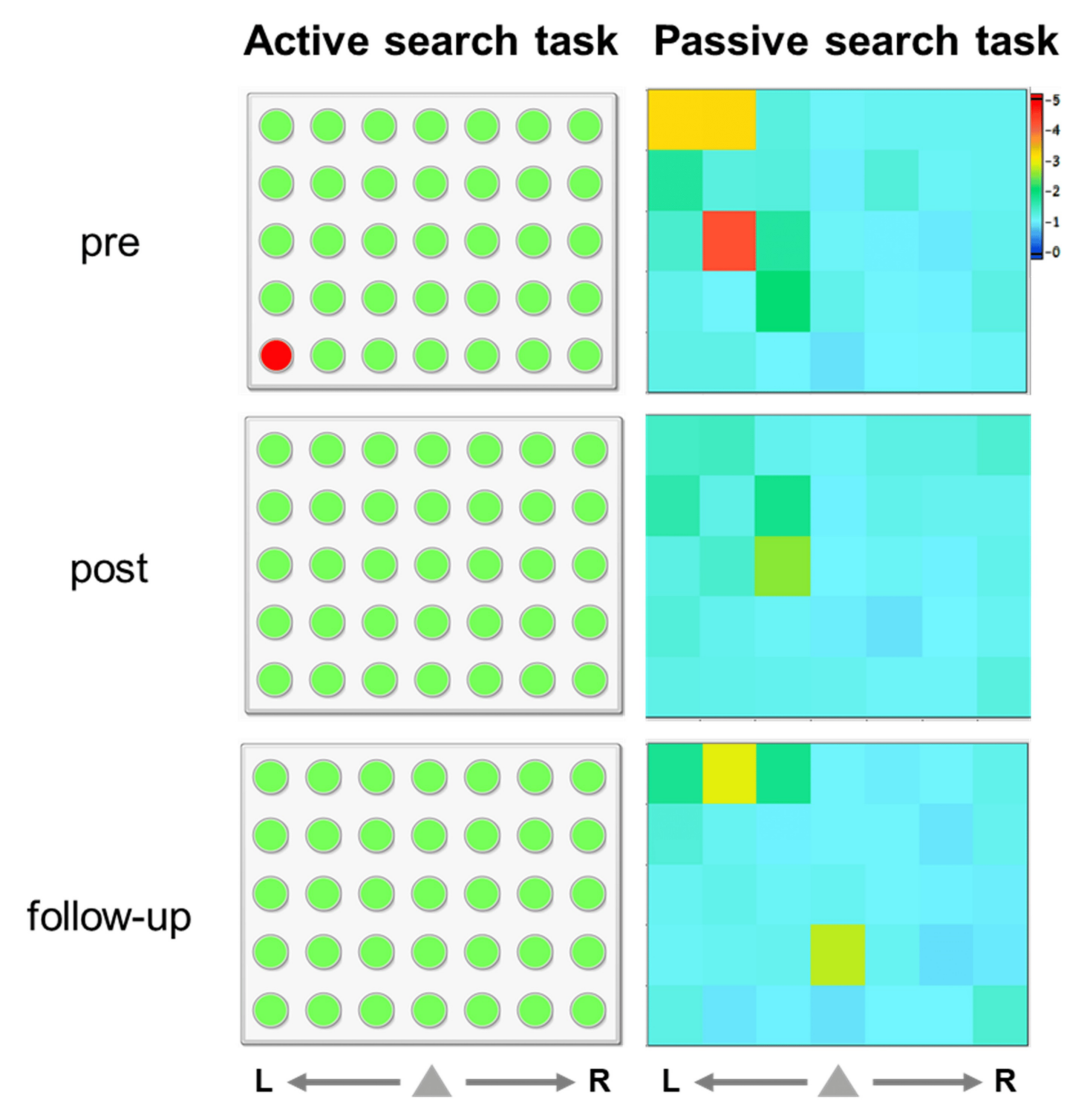
Results of @Attention. The left side shows the results of the active search task, while the right side shows those of the passive search task. In the active search task, the selected objects are displayed in green, repeatedly selected objects in red, and non-selected objects in black. The patient was able to select all objects at the pre, post, and follow-up. The results of the passive task are presented as a spatial distribution of response times to each object. Bluish color indicates faster reaction times (RTs), while reddish color indicates delayed RT or omission errors. After the intervention (post), omissions and delayed RTs on the left side (yellow and red) were reduced. Moreover, these improvements were maintained at the follow-up. Functionally, the patient no longer overlooked people or objects on his left side.

Figure 5 and Table 4 illustrate the results of @Attention. In the active search task (Figure 5, left side), all objects were selected (green) before and after the intervention, as well as during the follow-up period, without any omissions. The results of the passive task are presented as a spatial distribution of response times to each object (Figure 5, right side), where red indicates delayed RT and omission errors, in contrast to blue. The results indicate that omissions and delayed RTs were observed on the left side before the intervention (pre). However, these omissions and delayed RTs improved after the intervention (post). Furthermore, this improvement was maintained during the follow-up period. RT_mean_ were 1.36, 1.21, and 1.20 seconds at the pre-intervention, post-intervention, and follow-up phase, respectively, and LR_ratio_ values were 1.65, 1.24, and 1.26, respectively (Table 4). Although they were not fully normalized, the patient no longer overlooked people or objects on his left side during walking.

**Table 4:** Results of @Attention. RT_mean_ indicates mean reaction time during the task. LR_ratio_ is calculated as the average reaction time for objects on the left divided by that for objects on the right, excluding the central vertical column.

	Pre-intervention	Post-intervention	Follow-up
RT_mean _(s)	1.36	1.21	1.20
LR_ratio_	1.65	1.24	1.26

Although variability across trials or days was not systematically measured, improvements appeared consistent based on clinical observations.

## Discussion

This case study examined the combined effects of VST with TENS during walking on ADL in a subacute stroke patient with USN. The results suggested that the combination improved ADL in USN patients and further seemed effective in improving bottom-up attention.

In the current case, in addition to the slight improvement in BIT-c scores, CBS scores also improved after the intervention, and these improvements were maintained at the two-week follow-up. These changes indicate that neglect symptoms in ADL improved from a moderate to a mild level. As a result, he no longer overlooked people or objects on his left side and regained independent walking. Previous studies have reported that TENS on the left body can alleviate neglect symptoms in patients with USN [13,17,18]. For example, Vallar et al. [18] investigated the effects of TENS applied to the left posterior neck in patients with left USN and reported improvements in the letter cancellation tests. The underlying mechanism has been suggested to involve activation of the right cerebral hemisphere through TENS of the left body, leading to a centralization of the egocentric reference frame with respect to the external environment. Furthermore, Pitzalis et al. [17] found that TENS on the left posterior neck improved asymmetry in the latencies of visual evoked potentials between the left and right visual fields in patients with left USN, along with enhanced performance in line bisection tests. More importantly, the effects of TENS have been demonstrated to be more pronounced when combined with VST compared to TENS alone [19-21]. However, previous studies have primarily focused on improvements in pencil-and-paper tests, and the effectiveness of this combined intervention on ADL remains unclear. On the other hand, the combined effects of other interventions have been reported to improve ADL [30-32]. For example, Choi et al. [31] showed that combining prism adaptation (PA) with vibration applied to the left neck muscle improved not only scores on the Albert's test but also CBS scores in patients with USN, but the improvement in CBS scores with the combination of PA and vibration was similar to that observed when each intervention was applied individually. In addition, they reported that, in patients with acute USN, the combination of PA and TENS applied to the left wrist muscle led to improvements in the Albert's test and CBS scores, particularly in subitems 1, 2, 4, and 6, which are associated with non-ambulatory activities [30]. In contrast, Fukata et al. [33] reported improvements in CBS scores, including subitems 8 and 10, which are related to ambulatory activities, following standing and walking training that incorporated a laser pointer to enhance bottom-up attention in a patient with USN. Therefore, in the present case, the observed improvements in CBS scores may have resulted from the combination of VST, which involves walking while engaging top-down attention, and the bottom-up input provided by TENS. Taken together, improving ambulatory ADLs in patients with USN may require incorporating ADL tasks that are affected by neglect symptoms into the intervention. Specifically, training visuospatial attention while walking with bottom-up input may be necessary to improve neglect symptoms during walking. Nevertheless, given the single-case nature of this study and the lack of a control condition, it remains unclear whether the observed improvements were due to the combined effect of VST and TENS or to either intervention alone. Further intervention is warranted.

In the present case, the combination of VST with TENS also improved bottom-up attention. Since lesions in the ventral attention network have been reported to contribute to chronic USN [34], dysfunction of bottom-up attention appears to play a key role in its persistence. Thus, the observed improvements in bottom-up attention may be significant in preventing the chronicity of USN. To date, few reports have reported interventions that effectively enhance bottom-up attention, but one study demonstrated that TENS on the left body increased activity in the right hemisphere, including the supramarginal gyrus, a part of the ventral attention network, as well as the premotor and supplementary motor cortices, primary motor cortex, and somatosensory cortex, which was associated with improved bottom-up attention [35]. Therefore, in the present case, TENS of the left posterior neck muscle may have activated the right supramarginal gyrus, facilitating the recovery of bottom-up attention. However, since TENS and VST were applied simultaneously, it remains unclear whether the observed improvement was solely due to TENS or resulted from its combination with VST. Future studies should clarify this issue. Furthermore, it should also be noted that, in the absence of a control or single-intervention comparator, non-specific factors such as increased therapist attention or the novelty of the combined intervention may have contributed to the observed improvements.

Beyond the specific findings of this case, the results highlight the potential clinical value of training visuospatial attention in real-world contexts such as walking. For clinicians, this underscores the importance of addressing neglect symptoms within functionally relevant tasks. For researchers, the case supports the need to develop and evaluate integrated interventions that simultaneously target cognitive and motor recovery in patients with USN.

This case study has several limitations. First, as a single-case study, it remains unclear whether this combination is effective for patients with varying degrees of USN severity. Second, since only the combination of VST and TENS was implemented, the effect of each intervention alone is unclear. Therefore, caution is needed when interpreting the combined effects of VST with TENS. Third, the follow-up period was limited to two weeks after the intervention. Therefore, the long-term durability of the improvements observed remains unclear. It is also unknown whether continued or periodic maintenance training is necessary to sustain the functional gains.

## Conclusions

This case study suggests that combining VST with TENS during walking may be effective in improving both bottom-up attention and ambulation-related ADL in subacute stroke patients with USN. Notably, the patient demonstrated reduced left-sided omissions and regained independent walking, indicating meaningful functional gains that extend beyond the improvements typically captured by conventional paper-and-pencil assessments. These gains were maintained at follow-up, suggesting durability of the intervention effects over time. These findings highlight the potential clinical value of integrating cognitive and motor training in ecologically valid, real-world contexts. However, given the single-case nature of this study and the absence of a single-intervention comparator, caution is warranted in generalizing these results. Future research with controlled designs and larger sample sizes is needed to confirm the specific and combined effects of VST and TENS on USN.
